# Implant functionalization with mesoporous silica: A promising antibacterial strategy, but does such an implant osseointegrate?

**DOI:** 10.1002/cre2.389

**Published:** 2020-12-31

**Authors:** Katleen Vandamme, Karin Thevissen, Marcelo F. Mesquita, Ruxandra‐Gabriella Coropciuc, Jimoh Agbaje, Patrick Thevissen, Wander José da Silva, Jozef Vleugels, Kaat De Cremer, Evelien Gerits, Johan A. Martens, Jan Michiels, Bruno P. A. Cammue, Annabel Braem

**Affiliations:** ^1^ Department of Oral Health Sciences & Restorative Dentistry, Biomaterials—BIOMAT KU Leuven & University Hospitals Leuven Leuven Belgium; ^2^ Centre of Microbial and Plant Genetics KU Leuven Leuven Belgium; ^3^ Department of Prosthodontics and Periodontology, Piracicaba Dental School State University of Campinas Piracicaba Brazil; ^4^ Oral and Maxillo‐facial Surgery, Imaging & Pathology (OMFS‐IMPATH), Department of Oral Health Sciences & Department of Imaging and Pathology KU Leuven & University Hospitals Leuven Leuven Belgium; ^5^ Forensic Odontology, Department of Imaging and Pathology KU Leuven Leuven Belgium; ^6^ Department of Materials Engineering KU Leuven Leuven Belgium; ^7^ Centre of Plant Systems Biology Vlaams Instituut voor Biotechnologie Ghent Belgium; ^8^ Centre of Surface Chemistry and Catalysis KU Leuven Leuven Belgium; ^9^ VIB Center for Microbiology Flanders Institute for Biotechnology Leuven Belgium

**Keywords:** miniature, osseointegration, silicon dioxide, swine, titanium

## Abstract

**Objectives:**

New strategies for implant surface functionalization in the prevention of peri‐implantitis while not compromising osseointegration are currently explored. The aim of this in vivo study was to assess the osseointegration of a titanium‐silica composite implant, previously shown to enable controlled release of therapeutic concentrations of chlorhexidine, in the Göttingen mini‐pig oral model.

**Material and Methods:**

Three implant groups were designed: macroporous titanium implants (Ti‐Porous); macroporous titanium implants infiltrated with mesoporous silica (Ti‐Porous + SiO_2_); and conventional titanium implants (Ti‐control). Mandibular last premolar and first molar teeth were extracted bilaterally and implants were installed. After 1 month healing, the bone in contact with the implant and the bone regeneration in the peri‐implant gap was evaluated histomorphometrically.

**Results:**

Bone‐to‐implant contact and peri‐implant bone volume for Ti‐Porous versus Ti‐Porous + SiO_2_ implants did not differ significantly, but were significantly higher in the Ti‐Control group compared with Ti‐Porous + SiO_2_ implants. Functionalization of titanium implants via infiltration of a SiO_2_ phase into the titanium macropores does not seem to inhibit implant osseointegration. Yet, the importance of the implant macro‐design, in particular the screw thread design in a marginal gap implant surgery set‐up, was emphasized by the outstanding results of the Ti‐Control implant.

**Conclusions:**

Next‐generation implants made of macroporous Ti infiltrated with mesoporous SiO_2_ do not seem to compromise the osseointegration process. Such implant functionalization may be promising for the prevention and treatment of peri‐implantitis given the evidenced potential of mesoporous SiO_2_ for controlled drug release.

## 1 INTRODUCTION

Alterations in manufacturing processes and surgical techniques in implant therapy together with decreasing costs have rendered implant therapy for oral rehabilitation in partially and fully edentulous patients common. Additionally, high success levels (>95%) and good predictability have motivated patients to choose treatment with implants (Buser et al., 2017; Esposito et al., 2014; Naert et al., 2002; Quirynen et al., 2014).

Developments in implant dentistry were mainly related to enhance the rate of implant osseointegration as well as to address conditions with impaired bone quality at the bone‐implant interface (Joos et al., 2006; Nkenke & Fenner, 2006; Rupp et al., 2018). In order to accelerate the process of osseointegration and improve the strength of the established bone‐implant interface, implant surface characteristics have been investigated extensively (Meirelles et al., 2008; Pellegrini et al., 2018; Smeets et al., 2016; Wennerberg & Albrektsson, 2010). Optimization of the bone‐implant interface has been obtained by incorporating inorganic phases within or onto the titanium oxide layer, or by increasing the roughness (Cardoso et al., 2014; Dohan Ehrenfest et al., 2011; Kim et al., 2010; Wennerberg & Albrektsson, 2010). Despite that, patients still lose implants as a result of mechanical or biological complications. Summarized research findings point out two aspects as main causes of implant failure: occlusal overload and peri‐implantitis (Naert et al., 2012; Renvert & Quirynen, 2015; Schwarz et al., 2018).

Research is currently focusing on the development of effective treatment protocols to prevent or to bring to a halt the peri‐implantitis disease in patients receiving dental implants (Jepsen et al., 2015; Qin et al., 2018; Renvert & Polyzois, 2018; Sanz & Giannobile, 2018; Schwarz et al., 2015; Tonetti et al., 2015). Whereas the surface characteristics of the implant components influence not only the biocompatibility, but also the bacterial adhesion and colonization, the mechanisms restricting the formation of dense layers of micro‐organisms, so called biofilms, on these surfaces have been studied, with the rationale to decrease the initial bacterial adhesion and minimize the subsequent inflammation of the peri‐implant tissues (Lang et al., 2000; Mabboux et al., 2004; Norowski & Bumgardner, 2009; Sardin et al., 2004; Sennhenn‐Kirchner et al., 2009; Zhou et al., 2017). Among the possibilities to prevent or treat the increasing peri‐implantitis incidence and at the same time reduce the risk of antibiotic resistance development related to systemic drug administration are implant surface modifications that prevent adhesion of pathogens at the implant surface (e.g., nanostructured TiO_2_), the local administration of antimicrobial or antibiofilm drugs at the implant site (e.g., Zn‐ or Ag‐modified TiO_2_, chlorhexidine‐grafted titanium, chlorhexidine releasing hydroxyapatite coatings), or the physical removal (debridement) of biofilms (Campoccia et al., 2013; Hallström et al., 2012; Qian et al., 2012; Xu et al., 2017). In that context, we previously designed a dental implant composed of a porous titanium‐silica (Ti/SiO_2_) composite material and containing an internal reservoir that can be loaded with antimicrobial compounds. The antimicrobial compounds can diffuse in a controlled manner through the porous implant walls, thereby reducing microbial biofilm formation on the implant surface (Braem et al., 2015; De Cremer et al., 2017). Hence, these implants allow controlled release of compounds from the implant inside towards the tissues outside, favoring prevention and treatment of biofilm formation because of the local treatment while avoiding the occurrence or progression of peri‐implantitis. Proof‐of‐concept for the drug delivery functionality and concomitant antimicrobial activity of chlorhexidine released from this composite material has been established in vitro, and this is in a biofilm preventive as well as curative setup using the oral bacterial pathogen *Streptococcus mutans (*Braem et al., *2015* *)*. Yet, to further prove the usefulness of this novel material in the clinic, its influence on osseointegration with and without a functional drug release needs to be addressed as well.

Therefore, the present experiment evaluated the osseointegration potential of such porous Ti/SiO_2_ composite implant material in the absence of any drug release. Implant osseointegration was compared to the osseointegration of the macroporous Ti implant as such, as well as to a commercially pure dense Ti implant. The experiment was performed in the mandible of the Göttingen mini‐pig and implant osseointegration was evaluated by histomorphometry.

## 2 MATERIALS AND METHODS

### 2.1 Animals and surgery

Six 36‐month‐old female Göttingen mini‐pigs (Ellegaard, Göttingen minipigs A/S, Dalmose, Denmark) were used as experimental animals, due to their known physiology and similarities with human physiology (Stadlinger et al., 2012). The Ethics Committee for animal research of KU Leuven approved the study (P074/2015), accordingly to the regulations and guidelines of the Belgian animal welfare.

**FIGURE 1:**
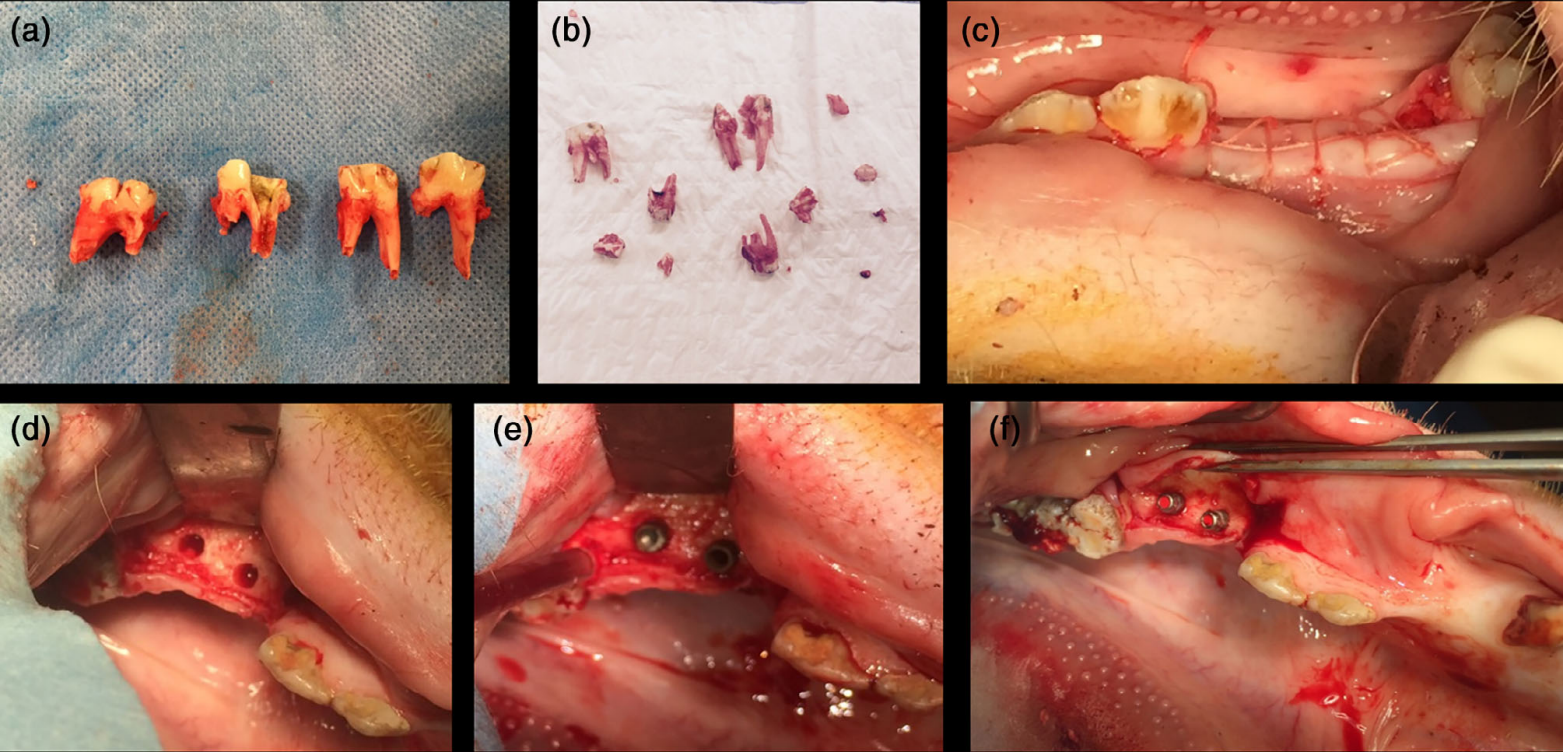
Clinical illustration of tooth extractions (a–c) and implant installation (d–f)

In order to allow implant installation, the last premolar and first molar on both sides of the lower jaw were extracted in a veterinary hospital under general anesthesia, following standard protocol (Figure 1). The animals were pre‐anesthetized with butyrophenone neuroleptic sedative (1 ml Stresnil^**®**^ i.m. Aurischter, Annadale, Australia), followed by anesthesia (1 ml/10 kg body weight i.v. of a cocktail of Zoletil^**®**^ 100 (Virbac, Barneveld, the Netherlands) with xylazine (Vexylan^**®**^, CEVA, Brussels, Belgium). Anesthesia maintenance was performed using gas anesthesia with 1.5% isoflurane (Isoflo^**®**^, Abott, QC, Canada). Post‐operatively, the animals received analgesia for 3 days by buprenorfine 0.005 mg/kg body weight i.m. (Temgesic^**®**^, Schering‐Plough, Brussels, Belgium) and antibiotic therapy during 5 days by amoxycillin (Duphamox^**®**^ LA, 0.1 ml/kg body weight i.m., Norbrook Laboratories Ltd., Newry, Ireland).

**FIGURE 2:**
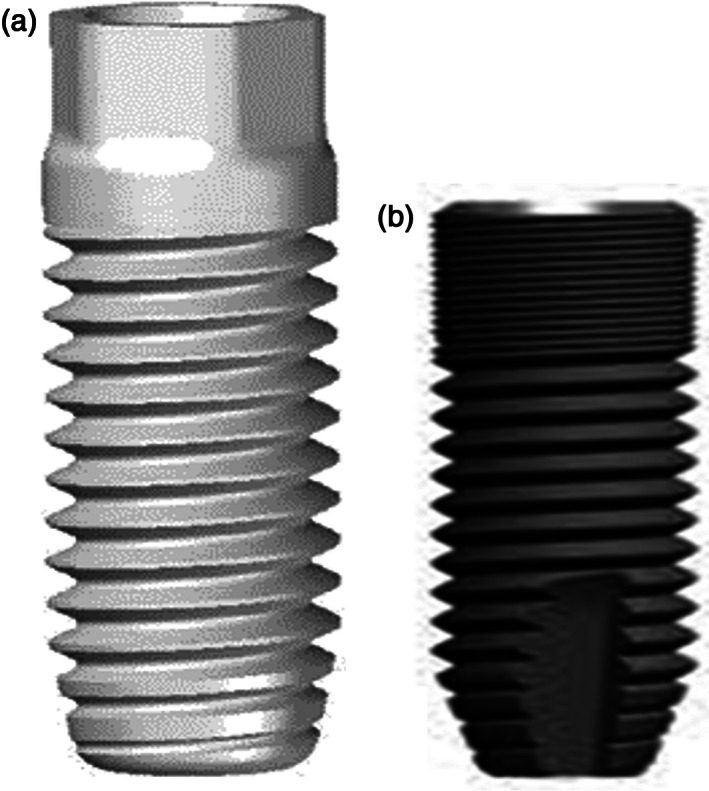
Design and dimensions of the implants. (a) Custom‐made porous titanium implant with or without mesoporous SiO_2_ diffusion barrier (group 1: Ti‐Porous; group 2: Ti‐Porous + SiO_2_), developed by MTM KU Leuven (Braem et al., 2015; De Cremer et al., 2017). (b) Commercial dense c.*p*. *titanium* implant (Astra Tech Osseospeed 3.5S, Denstply Sirona, Mölndal, Sweden, group 3: Ti‐Control). Scale bar: 1 mm

Three implant groups were designed as follows:

- Group 1 (*n* = 6): custom‐made porous titanium screw‐shaped implant (Ti‐Porous; Figure 2a) with dimensions of Ø 3.5 × 11 mm, fully interconnected open porosities of 30% and pore window size: 0.5–2.0 μm.
- Group 2 (*n* = 7): custom‐made porous titanium‐silica composite screw‐shaped implant (Ti‐Porous + SiO_2_; Figure 2a), with dimensions of Ø 3.5 × 11 mm. The composite material consists of a sol–gel derived mesoporous SiO_2_ diffusion barrier (pore diameter of 6.4 nm) integrated in the macroporous Ti load‐bearing structure.
- Group 3 (*n* = 10): commercial dense c.*p*. *titanium* screw‐shaped implants (Astra Tech OsseoSpeed 3.5 S, Dentsply Sirona, Mölndal, Sweden) with dimensions of Ø 3.5 × 8 mm (Ti‐Control; Figure 2b).

**FIGURE 3:**
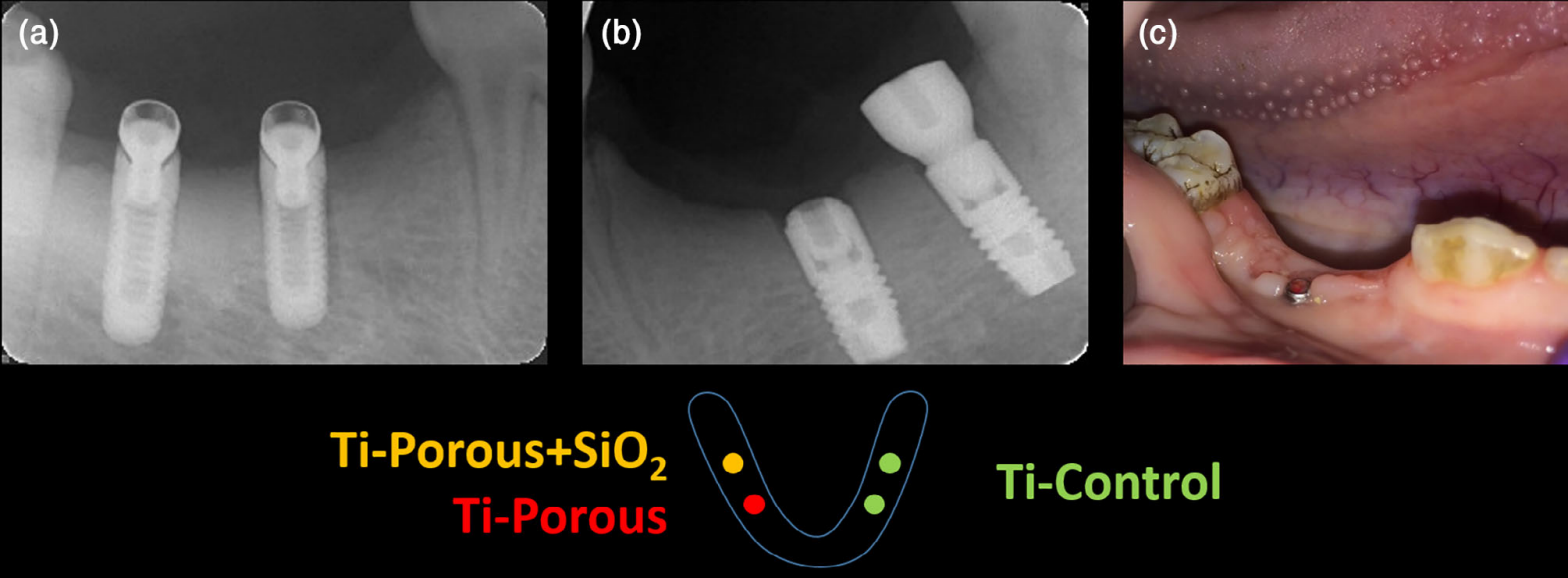
Evaluation of healing 1 month post‐implantation. (a and b) Representative intraoral X‐ray images of implant positioning of experimental and commercial implants, respectively. The schematic below indicates the position of the implants in the lower jaw bone. (c) Clinical observation of the implant site

Two months post‐extraction, healing was evaluated under sedation through clinical evaluation and intraoral digital radiographs (Soredex, Tuusula, Finland) with following parameters: 70 kV, 8 mA and 0.16 s. Four months post‐extraction, the installation of the implants was performed (identical analgesia, anesthesia and postoperative care protocol as was applied for the tooth extractions; Figure 3). Either one or two implants were installed in each quadrant. The allocation of the implant types to the operation sites was predefined taking into account an even distribution of the different implant types over the different positions (left vs. right quadrant; mesial versus distal position). After crestal incision, pilot holes were placed with precision drills at regulated speed. Wider drills were used to expand the implant holes (Ø 2.0 mm; Ø 3.0 mm; Ø 3.35 mm), followed by over‐preparation of the cavity using a Ø 3.85 mm bur. In this way, a circumferential gap of nearly 0.35 mm around all implants was created. Surgery was performed under abundant saline irrigation. The screw‐threaded part of the implants was placed equi‐osseus with the vestibular bone as a reference. Implants were inserted manually, without the use of a torque wrench. Because of the created defects around the implants, the implants did not have primary stability. Therefore, submersion underneath the gingiva of the implants was performed to allow proper healing. Given the height of the cover screws and the length of the implants of groups 1 and 2, and given the striving towards non‐tension primary closure, the buccal periosteum was relieved using a curved blade in the apical‐horizontal direction. Flaps and surgical wound margins were sutured. Furthermore, in order to avoid adverse mechanical forces affecting the implant osseointegration process because of mastication, a soft diet was applied from implant installation onwards.

After 1 month post‐implantation, healing was evaluated under sedation through clinical evaluation and intraoral digital radiographs, similarly as described above (Figure 3). Subsequently, the animals were euthanized by an intravascular injection of an embutramide‐mebenzoniumjodide‐tetracaine‐HCL solution (1 ml/5 kg body weight, T61^**®**^, Intervet, Mechelen, Belgium) into the ear vein until cardiac arrest occurred.

### 2.2 Sample processing and analysis

The implants with the surrounding jaw bone were harvested. The bone blocks were fixed by immersion in a CaCO_3−_buffered formalin solution (4%), dehydrated in an ascending series of ethanol concentrations for 18 days and embedded separately by infiltration of a benzoylperoxide (0.018%)–methylmetacrylate solution.

X‐rays of the bone samples with implants were taken to confirm the presence of the implants. These X‐rays were also used as a reference to define the cutting plane for separating the bone block into two halves containing each one single implant. Subsequently, the embedded bone blocks were mounted on a precision diamond saw (Leica SP 1600, Leica Microsystems, Nussloch, Germany). The cutting orientation was defined parallel to the implant and perpendicular to the jaw. Slices of approximately 700 μm thickness were obtained. Two or three sections per sample were selected for analysis. The sections were micro‐ground under running tap water and polished to a final thickness of 120–130 μm (Exact 400 CS grinding device, Exact Technologies Inc., Norderstedt, Germany). Finally, sections were stained with a combination of Stevenel's blue and Von Gieson's picrofushin red that allowed the visualization of mineralized bone (red) and demineralized tissue (blue‐green). Additionally, representative sections were analyzed by scanning electron microscopy (SEM), as described before (Braem et al., 2014). To this end and prior to histological staining, sections were ground with a 4000 grit SiC grinding paper. To produce a thin conductive film on the surface, the sections were coated with Au‐Pd with a sputtering device.

**FIGURE 4:**
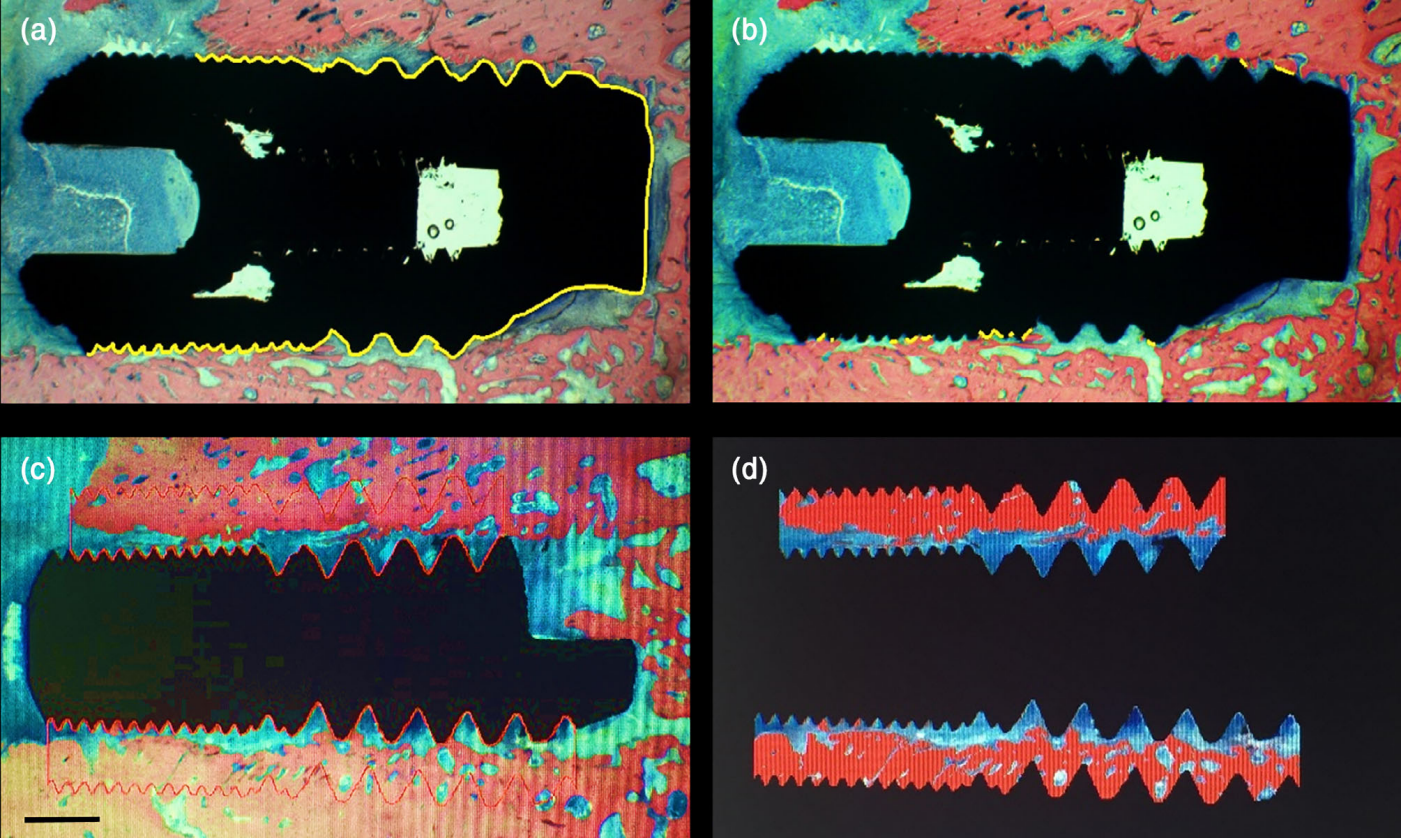
Illustration of the assessment of BIC and BV, shown for a commercial Ti‐Control implant. (a) Total length of the implant embedded into bone (indicated by the yellow line); (b) Summation of the lengths of the implant portions in direct contact with the bone (indicated by the yellow lines). Bone‐to‐implant contact (BIC) is then defined as the ratio of the latter over the total length of the implant. (c) A predetermined region of interest (ROI) for bone volume (BV) analysis is extending either 500 or 800 μm from the implant surface. For the current example, the ROI is extending 800 μm from the implant surface (delignated by the red line); (d) Subtracted image, solely visualizing the ROI. BV is then defined as the percentage of the ROI surface area occupied by bone. Staining with Stevenel's blue and Von Gieson's picrofushin red. Scale bar: 1 mm

Morphological and histomorphometric analyses were performed by light microscopy (Leitz Laborlux S, Wetzlar, Germany), with magnifications of 40×, 100× and 400×, or by SEM (XL30‐FEG, FEI, The Netherlands), performed under standard vacuum conditions at 10 mm working distance and 20 kV accelerating voltage using backscattered electron imaging. The histomorphometric evaluation was conducted with an ultra‐sensitive color video camera (AxioCam MRC5, Zeiss, Gottingen, Germany) mounted on an amplification glass with amplification of 10× (WILD Heerbrugg type MDG17, Switzerland) and using a color image analysis software (Axiovision 4.0, Zeiss) and a customized script particularly programmed for the needs of our analysis. The quantified variables were:

- Bone‐to‐implant contact (BIC), calculated as the proportion of the implant regions in direct contact with the bone over the total length of the implant embedded in bone tissue (Figure 4a,b).
- Bone volume (BV), calculated as the percentage area occupied by bone within the predetermined areas of interest. The BV was calculated for two areas of interest defined by two circumferential regions extending 500 and 800 μm from the implant surface, respectively. The rationale of this analysis was to identify to what distance from the implant surface the bone formation might have been influenced by the specific implant surface. To assess BV, the structures were semi‐automatically depicted using customized scripts running in the Axiovision images analysis software (Figure 4c,d). Values were expressed as a percentage of the total area of the region of interest.

### 2.3 Statistical analysis

Statistical analysis was performed using the software package Stat Plus, a statistical analysis program for MAC OS^**®**^ Version v6, for all the parameters. The *F*‐test for variance, and *t*‐test to compare the means, assuming equal (homoscedasticity) or unequal (heteroscedasticity) variances, were adopted. Means and standard deviations are given for each implant group. The results were verified using the one‐tailed *t*‐test (with 95% confidence intervals), with a significance level <0.05.

### 2.4 Ethics statement

The Ethics Committee for animal research of KU Leuven approved the study (P074/2015), accordingly to the regulations and guidelines of the Belgian animal welfare.

## 3 RESULTS

Wound healing, both post‐extraction and post‐implantation was uneventful. However, out of the 23 installed implants, two, two and one implants were lost in implant groups 1, 2 and 3, respectively, resulting in an overall implant osseointegration success of 78%. Per group, the success rate was 66.66%, 71.42% and 90%, respectively. Clinical inspection after 1 month of healing showed that some healing abutments had punctured the mucosa and were exposed to the oral cavity. This was observed *ad random* over the different groups. The gingiva and mucosa surrounding these exposed abutments was evaluated as healthy (Figure 3c).

**FIGURE 5:**
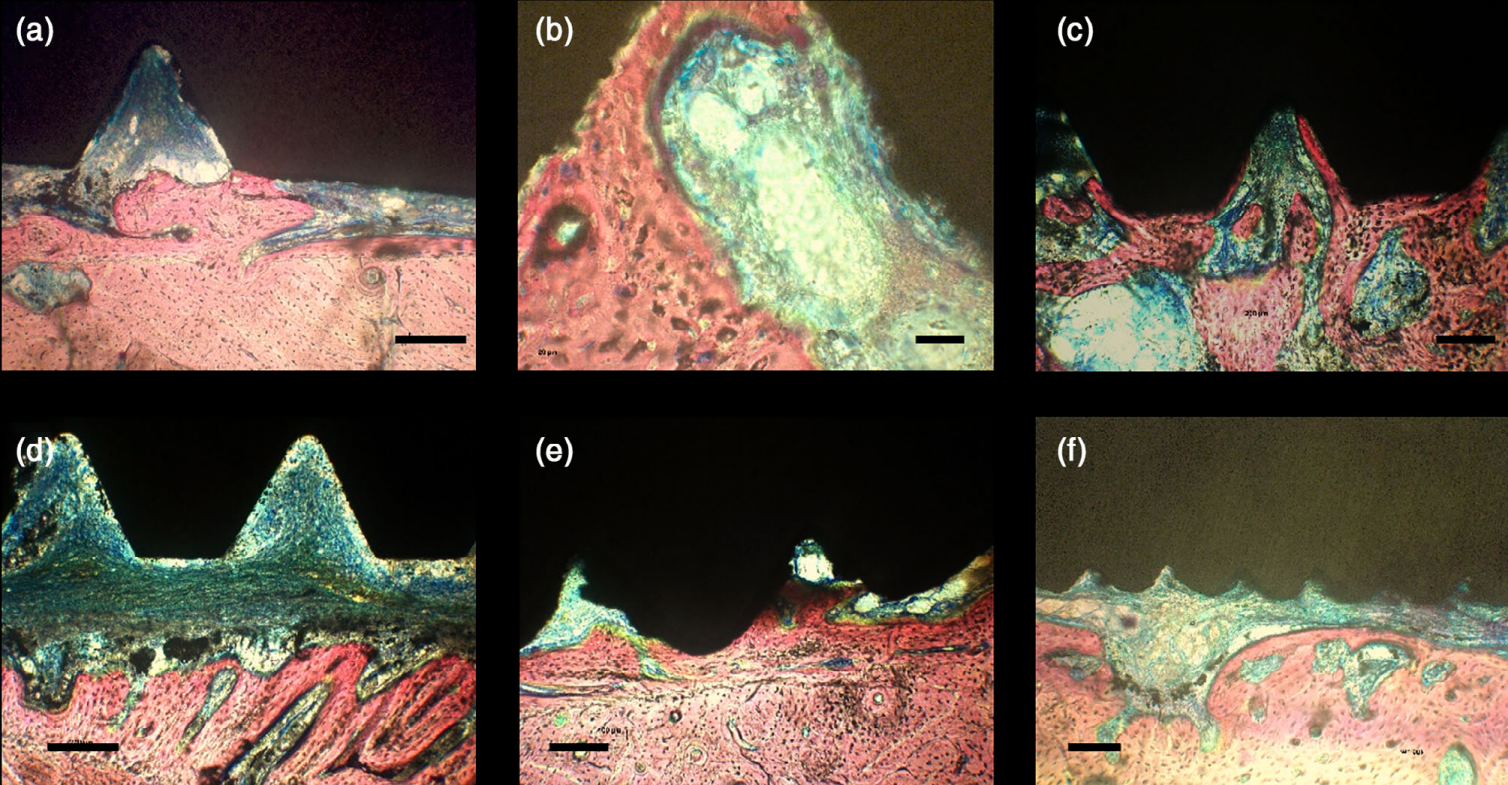
Representative histological illustrations of bone healing surrounding the implant (1 month healing). (a) Bone trabeculae arranged in a tridimensional network with some parts in contact with the implant surface. The gap between the implant and the host bone is partially filled with newly formed woven bone. Scale bar: 200 μm. (b) Initial signs of tissue organization including cubical osteoblast‐like cells interlaced into a network of recently released bone matrix. Scale bar: 50 μm. (c) Ridges of osteoid and woven bone and areas of immature marrow. Scale bar: 200 μm. (d) Bone trabeculae formation prevalently started from the lateral walls of the host bone and was directed towards the implant surface, with soft tissue interposed. Fibrous tissue is running parallel to the implant surface. Scale bar: 200 μm. (e) Bone apposition on the surface of the newly formed bone on the lateral wall of the defect. Scale bar: 200 μm. (f) Well‐organized tissue including the presence of trabecular mineralized bone connected to areas of non‐mineralized bone tissue. The presence of blood vessels suggests a higher level of tissue organization. Scale bar: 200 μm

**FIGURE 6:**
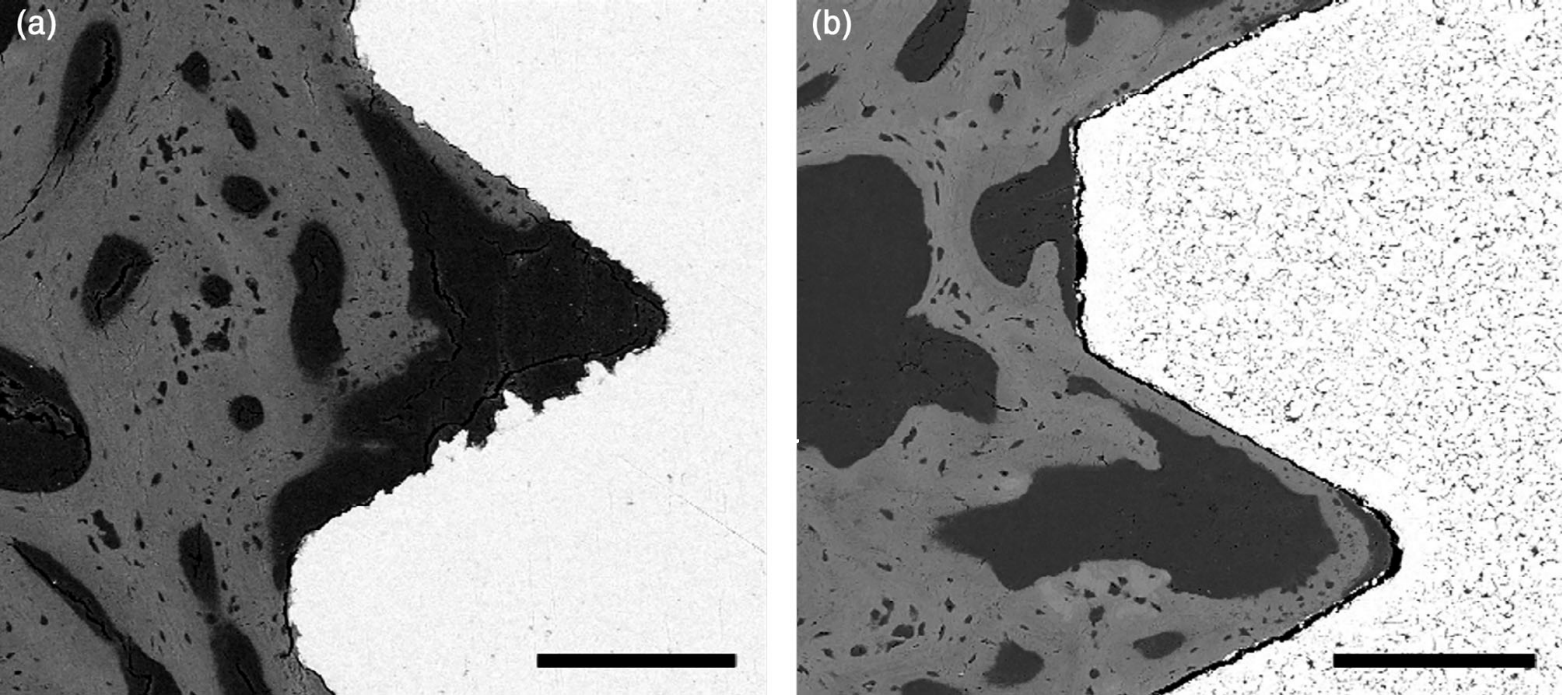
SEM images of the bone/implant interface (1 month healing). Bone‐to‐implant‐contact (BIC) could only be observed in limited zones for all implant conditions, but showed a close bone apposition for both (a) Ti‐Control and (b) experimental Ti‐Porous + SiO_2_ samples. Scale bar: 200 μm

Histological analysis did not reveal signs of infection. The filling of the defect with newly formed bone was incomplete after 1 month of healing in all specimens. Bone formation occurred from the base and the lateral walls of the defects. The presence of hematopoietic and pre‐osteoblastic cells could still be observed, as result of the ongoing bone regeneration process with the neoformation of bone trabeculae. Despite the fact that the implants have been installed without primary stability, BIC was observed in some samples though to a limited extent (Figure 5a–f). Yet, in the event of BIC, both Ti‐Control as well as experimental Ti‐Porous and Ti‐Porous + SiO_2_ samples showed a close bone apposition (Figure 6).

Table 1 summarizes the histomorphometric results for the different implant groups. Because several implants were lost upon exposure in the mini‐pig oral cavity, these data refer to a mean (±standard deviation) for four, five or nine implants for the Ti‐Porous, Ti‐Porous + SiO_2_ resp. Ti‐control groups. BIC did not differ significantly for Ti‐Porous versus Ti‐Porous+SiO_2_ implants. In contrast, significantly higher BIC values were measured for Ti‐Control implants compared with Ti‐Porous + SiO_2_ implants. Regarding the amount of bone regenerated in the peri‐implant gap at a distance of 500 and 800 μm from the implant surface (BV), no significant differences were observed for both gap distances between the Ti‐Porous and Ti‐Porous + SiO_2_ implants. Likewise BIC, Ti‐Control implants revealed significantly higher values for BV compared with Ti‐Porous + SiO_2_ implants.

**TABLE 1:** Mean values (%) and standard deviation of the histomorphometrical results for BIC and BV for the groups Ti‐Porous, Ti‐Porous + SiO_2_ and Ti‐Control (the data result from a total number of 4, 5 resp. 9 implants)

Parameter	Ti‐Porous	Ti‐Porous + SiO_2_	Ti‐Control	*p*‐value* for Ti‐Porous versus Ti‐Porous + SiO_2_	*p*‐value* for Ti‐Control versus Ti‐Porous + SiO_2_
BIC	1.15 (±2.62)	00.99 (±2.14)	17.92 (±10.55)	0.438	0.00001
BV 500	31.43 (±22.04)	24.60 (±18.52)	61.65 (±10.61)	0.273	2.764E−8
BV 800	46.30 (±22.14)	38.12 (±21.26)	70.10 (±7.45)	0.199	1.662E−7

Abbreviations: BIC, bone‐to‐implant contact; BV, bone volume, within a circumferential region extending either 500 or 800 μm from the implant surface.

^*^ *p* < 0.05.

## 4 DISCUSSION

Implant dentistry research has focused on the optimization of dental implants, aiming to obtain faster and better results by modulating the biological process of bone healing. This can be obtained through different ways of alterations, such as the implant surface topography (macro‐scale, micro‐scale and nano‐scale), the implant shape, and its chemical composition that can significantly affect protein adsorption and modulate cell adhesion, proliferation, differentiation and rate of mineralization in the early stages of bone healing. But implant modification is now gaining interest from another perspective, namely the perspective of preventing and tackling peri‐implantitis (Bumgardner et al., 2011; Johnson & García, 2015; Karoussis et al., 2018; Shahi et al., 2017; Shi et al., 2015). Braem and co‐workers established proof‐of‐concept for the drug delivery functionality of a mesoporous SiO_2_ barrier incorporated into a high‐strength macroporous Ti carrier (Braem et al., 2015; De Cremer et al., 2017). This novel and promising implant design possesses an internal reservoir which can be refilled, thereby ensuring controlled release of antimicrobial or antibiofilm compounds over sustained period of time.

Prior to evaluating the release of antibacterial compounds in a peri‐implantitis set‐up in vivo, evidence should first be provided that such an implant with specific surface characteristics osseointegrates as other implants do. Therefore, the present experiment was conducted for evaluation of the effect of SiO_2_ functionalization of a macroporous implant on the peri‐implant bone healing in vivo in the Göttingen mini‐pig, by comparing to an identical macroporous implant without modification with SiO_2_, and to the well‐documented c.*p*. *titanium* implant. It was hypothesized that the integration of SiO_2_ in the implant does not hamper implant osseointegration.

Regarding the osteotomy defect, filling of it with newly formed bone was incomplete in all specimens in this stage of healing of 1 month post‐implantation. These results are in agreement with previous work, investigating early healing periods in animal models (Botticelli et al., 2005; Rossi et al., 2012). Despite the absence of primary implant stability and thus the presence of implant (micro)movements, histology illustrated bone regeneration activity around the implants in all samples, regardless of the implant group analyzed, with the presence of blood cells, cubical osteoblast‐like cells interlaced into a network of recently released bone matrix and trabecular mineralizing and mineralized bone connected to the parent bone. Given the size of the peri‐implant defect and the short time span for healing, only limited zones with bone‐to‐implant contact were observed, but with close bone apposition for all samples.

Besides the bone tissue in contact with the implant, the amount of bone in the implant's surroundings contributes to the solid anchorage of an implant into the bone. For this reason, not only BIC but also BV in the closer (BV_500_) and broader (BV_800_) implant vicinity was calculated. No significant differences were found for BIC and BV_500/800_ for Ti‐Porous + SiO_2_ versus Ti‐Porous, suggesting that the functionalization of the Ti‐Porous implant with a mesoporous silica did not affect the peri‐implant bone response, neither negatively nor positively. Likewise, Inzunza and co‐workers illustrated that the viability and proliferation of the osteoblast‐like cells is not altered in contact with a mesoporous SiO_2_ coating on titanium (Inzunza et al., 2014). Moreover, in combination with bioactive glass, which is known to improve osseointegration around titanium (Braem et al., 2014), such coatings can also accelerate the formation of bone tissue in the implant periphery (Covarrubias et al., 2016). As the mesoporous SiO_2_ phase enables the controlled release of drugs into the implant surroundings (Braem et al., 2015) and as it was shown in the present study that SiO_2_ does not affect the osseointegration of the implant, such “hybrid” implant designs are promising for further exploration and use in a peri‐implantitis set‐up.

It should be taken into account that cell behavior and the consequent bone formation around an implant are not solely depending on specific isolated implant surface properties. The cellular response should be considered as a complex phenomenon that depends on several factors acting simultaneously, such as shape, chemical composition and macro/microtopography of the implant as well as the characteristics of the host bed. When comparing Ti‐Control with Ti‐Porous + SiO_2_ implants, it was observed that BIC and BVs values were significantly more elevated for the control group in this early healing stage. One of the important variables influencing and directing the healing process of bone in the early stages after implantation is the implant design, in the perspective of the osteoconductive property of titanium. The implant macro‐design differed between the control and the experimental group with respect to the screw thread design, neck design (microthreads) and apex design (bone window allowing tissue interlocking and providing anti‐rotational resistance to forces). These differences may have co‐influenced the biological and mechanical micro‐environment leading to a differential peri‐implant bone healing response. In an attempt to exclude the implant macro‐design differences between the control and experimental implants, a larger‐than‐clinically‐advised osteotomy was created. The implant cavity was prepared at wider dimensions compared to the implant diameter. This notwithstanding, it seemed that the implant neck microthread and body thread pitch, width and depth of the control implant were still favoring the bone‐to‐implant contact and bone volume in the implant's surrounding. In line with these thoughts, the difference between both groups (Ti‐Control versus Ti‐Porous + SiO_2_) was larger when analyzing BV_500_ compared to BV_800_. Repeating the present study while adopting longer healing periods and/or while using a more appropriate control implant design (which was not possible for the present implant prototype with an internal reservoir for drug release) is under consideration. Of note is that, in the classical surgical protocol of close bone‐to‐implant contact at installation, resident bone first resorbs prior to new bone formation (Rossi et al., 2014; Slaets et al., 2006; Slaets et al., 2007). A small gap around the implant during the early phases of healing may thus accelerate bone regeneration.

## 5 CONCLUSION

Overall, based on the findings and the limitations of the present study, it can be concluded that the functionalization of a macroporous titanium implant with SiO_2_ does not negatively affect the peri‐implant bone healing response, as observed in the jaw bone of Göttingen mini‐pigs. At the same time, the importance of the implant macro‐design, in particular the screw thread design in terms of number, width, depth and pitch, in a marginal gap implant surgery protocol was emphasized. Subsequent studies will explore the use of such Ti/SiO_2_ implants in preventing and treating peri‐implantitis given their potential for controlled release of antibiofilm compounds (Braem et al., 2015; De Cremer et al., 2017).

## CONFLICT OF INTEREST

The authors declare that there is no conflict of interest in this study.

## AUTHOR CONTRIBUTIONS

Study design: KV, KT, JV, JAM, JM, BPAC, AB. Study conduct: KV, MFM, GRC, JA, PT, WJdS, KDC, EG, AB. Data collection: KV, MFM, PT, WJdS, AB. Data analysis: KV, MFM, WJdS, AB. Data interpretation: KV, KT, MFM, JV, AB. Drafting manuscript: KV, KT, MFM, WJdS, KDC, AB. Approving final version of manuscript: all authors.

## Data Availability

The data that support the findings of this study are available on request from the corresponding author. The data are not publicly available due to privacy or ethical restrictions.

## References

[cre2389-bib-0001] Botticelli, D., Berglundh, T., Persson, L. G., & Lindhe, J. (2005). Bone regeneration at implants with turned or rough surfaces in self‐contained defects. An experimental study in the dog. Journal of Clinical Periodontology, 32, 448–455. 10.1111/j.1600-051X.2005.00693.x. 15842258

[cre2389-bib-0002] Braem, A., Chaudhari, A., Vivan Cardoso, M., Schrooten, J., Duyck, J., & Vleugels, J. (2014). Peri‐ and intra‐implant boné response to microporous Ti coatings with surface modification. Acta Biomaterialia, 10, 986–995. 10.1016/j.actbio.2013.10.017. 24161385

[cre2389-bib-0003] Braem, A., De Cremer, K., Delattin, N., De Brucker, K., Neirinck, B., Vandamme, K., Martens, J. A., Michiels, J., Vleugels, J., Cammue, B. P. A., & Thevissen, K. (2015). Novel anti‐infective implant substrates: Controlled release of antibiofilm compounds from mesoporous silica‐containing macroporous titanium. Colloids and Surfaces B: Biointerfaces, 126, 481–488. 10.1016/j.colsurfb.2014.12.054. 25601097

[cre2389-bib-0004] Bumgardner, J. D., Adatrow, P., Haggard, W. O., & Norowski, P. A. (2011). Emerging antibacterial biomaterial strategies for the prevention of peri‐implant inflammatory diseases. International Journal of Oral and Maxillofacial Implants, 26, 553–560. 21691602

[cre2389-bib-0005] Buser, D., Sennerby, L., & De Bruyn, H. (2017). Modern implant dentistry based on osseointegration: 50years of progress, current trends and open questions. Periodontolology 2000, 73(1), 7–21. 10.1111/prd.12185. 28000280

[cre2389-bib-0006] Campoccia, D., Montanaro, L., & Arciola, C. R. (2013). A review of the biomaterials technologies for infection‐resistant surfaces. Biomaterials, 34, 8533–8554. 10.1016/j.biomaterials.2013.07.089. 23953781

[cre2389-bib-0007] Cardoso, M. V., Chaudhari, A., Yoshida, Y., Van Meerbeek, B., Naert, I., & Duyck, J. (2014). Bone tissue response to implant surfaces functionalized with phosphate‐containing polymers. Clinical Oral Implants Research, 25, 91–100. 10.1111/clr.12053. 23039076

[cre2389-bib-0008] Covarrubias, C., Mattmann, M., Von Marttens, A., Caviedes, P., Arriagada, C., Valenzuela, F., Rodríguez, F. P., & Corral, C. (2016). Osseointegration properties of titanium dental implants modified with a nanostructured coating based on ordered porous silica and bioactive glass nanoparticles. Applied Surface Science, 363, 286–295. 10.1016/j.apsusc.2015.12.022.

[cre2389-bib-0009] De Cremer, K., Braem, A., Gerits, E., De Brucker, K., Vandamme, K., Martens, J. A., Michiels, J., Vleugels, J., Cammue, B. P. A., & Thevissen, K. (2017). Controlled release of chlorhexidine from a mesoporous silica‐containing macroporous titanium dental implant prevents microbial biofilm formation. European Cell & Materials, 33, 13–27. 10.22203/eCM.v033a02. 28076651

[cre2389-bib-0010] Dohan Ehrenfest, D. M., Vazquez, L., Park, Y. J., Sammartino, G., & Bernard, J. P. (2011). Identification card and codification of the chemical and morphological characteristics of 14 dental implant surfaces. Journal of Oral Implantology, 37, 525–542. 10.1563/AAID-JOI-D-11-00080. 21728785

[cre2389-bib-0011] Esposito, M., Ardebili, Y., & Worthington, H. V. (2014). Interventions for replacing missing teeth: Different types of dental implants. The Cochrane Database of Systematic Reviews, 22, CD003815. 10.1002/14651858.CD003815.pub4. 25048469

[cre2389-bib-0012] Hallström, H., Persson, G. R., Lindgren, S., Olofsson, M., & Renvert, S. (2012). Systemic antibiotics and debridement of peri‐implant mucositis. A randomized clinical trial. Journal of Clinical Periodontology, 39, 574–581. 10.1111/j.1600-051X.2012.01884.x 22571225

[cre2389-bib-0013] Inzunza, D., Covarrubias, C., Von Marttens, A., Leighton, Y., Carvajal, J. C., Valenzuela, F., Díaz‐Dosque, M., Méndez, N., Martínez, C., Pino, A. M., Rodríguez, J. P., Cáceres, M., & Smith, P. (2014). Synthesis of nanostructured porous silica coatings on titanium and their cell adhesive and osteogenic differentiation properties. Journal of Biomedical Materials Research. Part A., 102, 37–48. 10.1002/jbm.a.34673. 23568757

[cre2389-bib-0014] Jepsen, S., Berglundh, T., Genco, R., Aass, A. M., Demirel, K., Derks, J., Figuero, E., Giovannoli, J. L., Goldstein, M., Lambert, F., Ortiz‐Vigon, A., Polyzois, I., Salvi, G. E., Schwarz, F., Serino, G., Tomasi, C., & Zitzmann, N. U. (2015). Primary prevention of peri‐implantitis: Managing peri‐implant mucositis. Journal of Clinical Periodontology, 42, S152–S157. 10.1111/jcpe.12369. 25626479

[cre2389-bib-0015] Johnson, C. T., & García, A. J. (2015). Scaffold‐based anti‐infection strategies in bone repair. Annals of Biomedical Engineering, 43, 515–528. 10.1007/s10439-014-1205-3. 25476163 PMC4380521

[cre2389-bib-0016] Joos, U., Wiesmann, H. P., Szuwart, T., & Meyer, U. (2006). Mineralization at the interface of implants. International Journal of Oral and Maxillofacial Surgery, 35, 783–790. 10.1016/j.ijom.2006.03.013. 16697141

[cre2389-bib-0017] Karoussis, I. K., Kyriakidou, K., Papaparaskevas, J., Vrotsos, I. A., Simopoulou, M., & Kotsakis, G. A. (2018). Osteostimulative calcium phosphosilicate biomaterials partially restore the cytocompatibility of decontaminated titanium surfaces in a peri‐implantitis model. Journal of Biomedical Materials Research. Part B Applied Biomaterials, 106(7), 2645–2652. 10.1002/jbm.b.34081. 29405560

[cre2389-bib-0018] Kim, S. J., Kim, M. R., Rim, J. S., Chung, S. M., & Shin, S. W. (2010). Comparison of implant stability after different implant surface treatments in dog bone. Journal of Applied Oral Science, 18, 415–420. 10.1590/S1678-77572010000400016. 20835579 PMC5349075

[cre2389-bib-0019] Lang, N. P., Wilson, T. G., & Corbet, E. F. (2000). Biological complications with dental implants: Their prevention, diagnosis and treatment. Clinical Oral Implants Research, 11, 146–155. 10.1034/j.1600-0501.2000.011S1146.x. 11168263

[cre2389-bib-0020] Mabboux, F., Ponsonnet, L., Morrier, J. J., Jaffrezic, N., & Barsotti, O. (2004). Surface free energy and bacterial retention to saliva‐coated dental implant materials ‐ an in vitro study. Colloids and Surfaces. B: Biointerfaces, 39, 199–205. 10.1016/j.colsurfb.2004.08.002. 15555904

[cre2389-bib-0021] Meirelles, L., Currie, F., Jacobsson, M., Albrektsson, T., & Wennerberg, A. (2008). The effect of chemical and nanotopographical modifications on the early stages of osseointegration. International Journal of Oral and Maxillofacial Implants, 23, 641–647. 18807559

[cre2389-bib-0022] Naert, I., Duyck, J., & Vandamme, K. (2012). Occlusal overload and bone/implant loss. Clinical Oral Implants Research, 23, 95–107. 10.1111/j.1600-0501.2012.02550.x. 23062133

[cre2389-bib-0023] Naert, I., Koutsikakis, G., Duyck, J., Quirynen, M., Jacobs, R., & van Steenberghe, D. (2002). Biologic outcome of implant‐supported restorations in the treatment of partial edentulism. Part I: A longitudinal clinical evaluation. Clinical Oral Implants Research, 13, 381–389. 10.1034/j.1600-0501.2002.130406.x. 12175375

[cre2389-bib-0024] Nkenke, E., & Fenner, M. (2006). Indications for immediate loading of implants and implant success. Clinical Oral Implants Research, 17, 19–34. 10.1111/j.1600-0501.2006.01348.x. 16968379

[cre2389-bib-0025] Norowski, P. A., & Bumgardner, J. D. (2009). Biomaterial and antibiotic strategies for peri‐implantitis: A review. Journal of Biomedical Materials Research. Part B Applied Biomaterials, 88, 530–543. 10.1002/jbm.b.31152. 18698626

[cre2389-bib-0026] Pellegrini, G., Francetti, L., Barbaro, B., & Del Fabbro, M. (2018). Novel surfaces and osseointegration in implant dentistry. Journal of Investigative and Clinical Dentistry, 4, e12349. 10.1111/jicd.12349. 29971928

[cre2389-bib-0027] Qian, J., Wennerberg, A., & Albrektsson, T. (2012). Reasons for marginal bone loss around oral implants. Clinical Implant Dentistry and Related Research, 14, 792–807. 10.1111/cid.12014. 23198697

[cre2389-bib-0028] Qin, S., Xu, K., Nie, B., Ji, F., & Zhang, H. (2018). Approaches based on passive and active antibacterial coating on titanium to achieve antibacterial activity. Journal of Biomedical Materials Research, 106(9), 2531–2539. 10.1002/jbm.a.36413. 29603857

[cre2389-bib-0029] Quirynen, M., Herrera, D., Teughels, W., & Sanz, M. (2014). Implant therapy: 40 years of experience. Periodontology 2000, 66, 7–12. 10.1111/prd.12060. 25123758

[cre2389-bib-0030] Renvert, S., & Polyzois, I. (2018). Treatment of pathologic peri‐implant pockets. Periodontology 2000, 76, 180–190. 10.1111/prd.12149. 29239086

[cre2389-bib-0031] Renvert, S., & Quirynen, M. (2015). Risk indicators for peri‐implantitis. A narrative review. Clinical Oral Implants Research, 26, 15–44. 10.1111/clr.12636. 26385619

[cre2389-bib-0032] Rossi, F., Botticelli, D., Pantani, F., Pereira, F. P., Salata, L. A., & Lang, N. P. (2012). Bone healing pattern in surgically created circumferential defects around submerged implants: An experimental study in dog. Clinical Oral Implants Research, 23, 41–48. 10.1111/j.1600-0501.2011.02170.x. 21443594

[cre2389-bib-0033] Rossi, F., Lang, N. P., De Santis, E., Morelli, F., Favero, G., & Botticelli, D. (2014). Bone‐healing pattern at the surface of titanium implants: An experimental study in the dog. Clinical Oral Implants Research, 25, 124–131. 10.1111/clr.12097. 23289845

[cre2389-bib-0034] Rupp, F., Liang, L., Geis‐Gerstorfer, J., Scheideler, L., & Hüttig, F. (2018). Surface characteristics of dental implants: A review. Dental Materials, 34, 40–57. 10.1016/j.dental.2017.09.007. 29029850

[cre2389-bib-0035] Sanz, M., & Giannobile, W. V. (2018). Soft and hard tissue augmentation procedures for promotion of peri‐implant health and aesthetics. Clinical Oral Implants Research, 29, 4–6. 10.1111/clr.13102. 29498130

[cre2389-bib-0036] Sardin, S., Morrier, J. J., Benay, G., & Barsotti, O. (2004). In vitro streptococcal adherence on prosthetic and implant materials. Interactions with physicochemical surface properties. Journal of Oral Rehabilitation, 31, 140–148. 10.1046/j.0305-182X.2003.01136.x. 15009598

[cre2389-bib-0037] Schwarz, F., Derks, J., Monje, A., & Wang, H. L. (2018). Peri‐implantitis. Journal of Periodontology, 89, S267–S290. 10.1002/JPER.16-0350. 29926957

[cre2389-bib-0038] Schwarz, F., Schmucker, A., & Becker, J. (2015). Efficacy of alternative or adjunctive measures to conventional treatment of peri‐implant mucositis and peri‐implantitis: A systematic review and meta‐analysis. International Journal of Implant Dentistry, 1, 22. 10.1186/s40729-015-00231. 27747644 PMC5005629

[cre2389-bib-0039] Sennhenn‐Kirchner, S., Wolff, N., Klaue, S., Mergeryan, H., & Borg‐von Zepelin, M. (2009). Decontamination efficacy of antiseptic agents on in vivo grown biofilms on rough titanium surfaces. Quintessence International, 40, e80–e88. 19898707

[cre2389-bib-0040] Shahi, R. G., Albuquerque, M. T. P., Münchow, E. A., Blanchard, S. B., Gregory, R. L., & Bottino, M. C. (2017). Novel bioactive tetracycline‐containing electrospun polymer fibers as a potential antibacterial dental implant coating. Odontology, 105, 354–363. 10.1007/s10266-016-0268-z. 27585669 PMC5539771

[cre2389-bib-0041] Shi, J., Liu, Y., Wang, Y., Zhang, J., Zhao, S., & Yang, G. (2015). Biological and immunotoxicity evaluation of antimicrobial peptide‐loaded coatings using a layer‐by‐layer process on titanium. Scientific Reports, 9, 5, 16336. 10.1038/srep16336. PMC4637835 26548760

[cre2389-bib-0042] Slaets, E., Carmeliet, G., Naert, I., & Duyck, J. (2006). Early cellular responses in cortical bone healing around unloaded titanium implants: An animal study. Journal of Periodontology, 77, 1015–1024. 16734577 10.1902/jop.2006.050196

[cre2389-bib-0043] Slaets, E., Carmeliet, G., Naert, I., & Duyck, J. (2007). Early trabecular bone healing around titanium implants: A histologic study in rabbits. Journal of Periodontology, 78, 510–517. 10.1902/jop.2006.050196. 17335375

[cre2389-bib-0044] Smeets, R., Stadlinger, B., Schwarz, F., Beck‐Broichsitter, B., Jung, O., Precht, C., Kloss, F., Gröbe, A., Heiland, M., & Ebker, T. (2016). Impact of dental implant surface modifications on osseointegration. BioMed Research International, 2016, 6285620. 10.1155/2016/6285620. 27478833 PMC4958483

[cre2389-bib-0045] Stadlinger, B., Pourmand, P., Locher, M. C., & Schulz, M. C. (2012). Systematic review of animal models for the study of implant integration, assessing the influence of material, surface and design. Journal of Clinical Periodontology, 39, 28–36. 10.1111/j.1600-051X.2011.01835.x. 22533945

[cre2389-bib-0046] Tonetti, M. S., Chapple, I. L., Jepsen, S., & Sanz, M. (2015). Primary and secondary prevention of periodontal and peri‐implant diseases: Introduction to, and objectives of the 11th European Workshop on Periodontology consensus conference. Journal of Clinical Periodontology, 42, S1–S4. 10.1111/jcpe.12382. 25683242

[cre2389-bib-0047] Wennerberg, A., & Albrektsson, T. (2010). On implant surfaces: A review of current knowledge and opinions. International Journal of Oral and Maxillofacial Implants, 25, 63–74. 20209188

[cre2389-bib-0048] Xu, G., Shen, X., Dai, L., Ran, Q., Ma, P., & Cai, K. (2017). Reduced bacteria adhesion on octenidine loaded mesoporous silica nanoparticles coating on titanium substrates. Materials Science and Engineering, 70, 386–395. 10.1016/j.msec.2016.08.050. 27770907

[cre2389-bib-0049] Zhou, W., Jia, Z., Xiong, P., Yan, J., Li, Y., Li, M., Cheng, Y., & Zheng, Y. (2017). Bioinspired and biomimetic AgNPs/gentamicin‐embedded silk fibroin coatings for robust antibacterial and osteogenetic applications. ACS Applied Materials & Interfaces, 9, 25830–25846. 10.1021/acsami.7b06757. 28731325

